# Supplementary material for: Active site specificity profiling datasets of matrix metalloproteinases (MMPs) 1, 2, 3, 7, 8, 9, 12, 13 and 14
Source: Data Brief. 2016 Feb 22;7:299–310. doi: 10.1016/j.dib.2016.02.036 (PMC4777984; doi:10.1016/j.dib.2016.02.036)
Supplement: Supplementary file 10 — Supplementary material [file mmc10.zip › WebPICS_hMMP1_T_1%/P2.html]

 

PICS results


|  |  |
| --- | --- |
| **P2\_A**  23 in 277 sites   8.3 %    effects > 10 perc. pnts.  (vice-versa in brackets)  P2prime\_Q: 12.0 (18.4)   P3prime\_V: 18.2 (19.0) |  |
| **P2\_C**  11 in 277 sites   4.0 %    effects > 10 perc. pnts.  (vice-versa in brackets)  P1\_C: 16.0 (29.3) |  |
| **P2\_D**  24 in 277 sites   8.7 %    effects > 10 perc. pnts.  (vice-versa in brackets)  P2prime\_T: 14.3 (19.1) |  |
| **P2\_L**  37 in 277 sites   13.4 %    effects > 10 perc. pnts.  (vice-versa in brackets)  P3\_C: 11.9 (36.6)   P3prime\_D: 18.5 (22.1)   P3prime\_E: 12.9 (19.9) |  |
| **P2\_M**  10 in 277 sites   3.6 %    effects > 10 perc. pnts.  (vice-versa in brackets)  P1\_F: 14.9 (10.7)   P1prime\_V: 32.1 (14.6)   P2prime\_M: 14.9 (10.7) |  |
| **P2\_Q**  34 in 277 sites   12.3 %    effects > 10 perc. pnts.  (vice-versa in brackets)  P2prime\_I: 12.7 (14.4) |  |
| **P2\_S**  25 in 277 sites   9.0 %    effects > 10 perc. pnts.  (vice-versa in brackets)  P1\_G: 11.0 (11.0)   P1prime\_M: 11.7 (12.7) |  |
| **P2\_W**  3 in 277 sites   1.1 %    effects > 10 perc. pnts.  (vice-versa in brackets)  P2prime\_H: 62.0 (14.3) |  |
